# EHR2Vec: Representation Learning of Medical Concepts From Temporal Patterns of Clinical Notes Based on Self-Attention Mechanism

**DOI:** 10.3389/fgene.2020.00630

**Published:** 2020-06-29

**Authors:** Li Wang, Qinghua Wang, Heming Bai, Cong Liu, Wei Liu, Yuanpeng Zhang, Lei Jiang, Huji Xu, Kai Wang, Yunyun Zhou

**Affiliations:** ^1^Department of Medical Informatics, Medical School, Nantong University, Nantong, China; ^2^Research Center for Intelligence Information Technology, Nantong University, Nantong, China; ^3^Department of Biomedical Informatics, Columbia University, New York, NY, United States; ^4^Department of Rheumatology and Immunology, Changzheng Hospital, The Second Military Medical University, Shanghai, China; ^5^Beijing Tsinghua Chang Gung Hospital, School of Clinical Medicine, Tsinghua University, Beijing, China; ^6^Peking-Tsinghua Center for Life Sciences, Tsinghua University, Beijing, China; ^7^Raymond G. Perelman Center for Cellular and Molecular Therapeutics, Children's Hospital of Philadelphia, Philadelphia, PA, United States; ^8^Department of Pathology and Laboratory Medicine, Perelman School of Medicine, University of Pennsylvania, Philadelphia, PA, United States

**Keywords:** natural language processing, representation learning, electronic health record, unstructured clinical notes, word vector

## Abstract

Efficiently learning representations of clinical concepts (i. e., symptoms, lab test, etc.) from unstructured clinical notes of electronic health record (EHR) data remain significant challenges, since each patient may have multiple visits at different times and each visit may contain different sequential concepts. Therefore, learning distributed representations from temporal patterns of clinical notes is an essential step for downstream applications on EHR data. However, existing methods for EHR representation learning can not adequately capture either contextual information per-visit or temporal information at multiple visits. In this study, we developed a new vector embedding method called EHR2Vec that can learn semantically-meaningful representations of clinical concepts. EHR2Vec incorporated the self-attention structure and showed its utility in accurately identifying relevant clinical concept entities considering time sequence information from multiple visits. Using EHR data from systemic lupus erythematosus (SLE) patients as a case study, we showed EHR2Vec outperforms in identifying interpretable representations compared to other well-known methods including Word2Vec and Med2Vec, according to clinical experts' evaluations.

## Introduction

In the field of clinical natural language processing (NLP), deep learning (DL) techniques outperform other NLP methods in many tasks, such as information extraction, named entity detection, and relationship assignment, etc. In DL-based NLP approaches, learning representative features is the critical step for the following analysis, such as classifications, clustering, and more. According to a recent review by Wu et al. (2020), among 1,737 clinical NLP articles, 74.1% of which used the DL-based Word2Vec method till 2018. Word2Vec is an unsupervised feature extraction method for representation learning, which converts word to numerical embedding by mapping each of the word tokens into high-dimensional vector space (Mikolov et al., 2013). Words that are closely related in certain conditions will be clustered together and share short distance in high-dimensional semantic space.

There are two types of DL architectures in Word2Vec model, CBOW, and Skip-gram. However, Word2Vec has limitations in capturing contextual information globally when analyzing clinical notes of electronic health records (EHR) (Mikolov et al., 2013). First, it scans the nearby relationship of each center word by a sliding window with a fixed size, yet considers each word in the window with equal importance; second, it does not incorporate temporal relationships of events on the same patients over time. In real-world EHR data, identifying representative medical entities is more complicated since the sequential relationship among entities per visit and temporal relationship among multiple visits need to be considered. For example, each patient has multiple visits for different reasons mapping to different visiting events at different time points; and the intervals of the patient's multiple medical events may vary from a few days to several months. More issues complicated these problems are that entities from different medical categories (i.e., diagnosis, medication, and procedures) often constitute disordered sequential collections and ignored long-range semantic dependencies.

To overcome the challenges of handling temporal issues, Med2Vec was proposed by Choi et al. (2016) to learn medical entity representations for EHR data at the visit level (Choi et al., 2016). Med2Vec essentially adopted the Word2Vec structure but has two layers; the first layer is to capture the relations between medical entities within a visit, and the second layer is to capture the relations between medical visit sequences. However, Med2Vec does not overcome the limitations of Word2Vec that considers the surrounding words within a window to be equally important, which makes the representative learning less efficient and inaccurate. In real-world EHR data, the impact of its nearby terms differs relative to the center word.

In this project, we proposed a new representation learning method for embedding medical entities for EHR data, called EHR2Vec. The EHR2Vec model incorporated a self-attention mechanism to learn important representations by updating values of the context words as a whole per visiting event. The self-attention algorithm, which is a DL-based method, was initially used in image processing (Vaswani et al., 2017). Instead of considering every part of the entire image equally important, it focuses on a specific portion while down weighting other parts of an image, which greatly improves the learning accuracy for the point of interest. Similar to NLP task in general documents, each patient's EHR data from multiple visits can be considered as a document composed of many sentences, while each visit can be considered as a sentence composed of many medical entity tokens. Because of the information heterogeneity of medical entities, we grouped them into four categories: medication, diagnosis, symptom, and lab test. Since time sequence information is an important factor in finding the most relevant representations at a certain time point, we sorted the medical events of each patient in the order of time to improve learning accuracy.

Compared to existing methods, EHR2Vec method has following characteristics: (1) it applies self-attention algorithm with multi-headed design to identify important global representations at visit level, which greatly improves embedding accuracy compared to previous word embedding methods; (2) it enables more accurate symptom detections in a temporal order, which can be used to facilitate predictions of disease progression trajectories. In the current study, we applied EHR2Vec on Systemic lupus erythematosus (SLE) data and compared the results with clinicians' manual interpretations. The results of the experiment indicated that EHR2Vec's high-dimensional embedding features are interpretable and consistent with clinician's opinions.

## Materials and Methods

### Description of Experimental Data

Since SLE is a chronic autoimmune disease which can gradually develop to multiple comorbidities from mild to serious as time elapses, we used SLE as a apt disease on which to test our approach. SLE involves multiple organs and systems throughout the body, often accompanied by various comorbidities, of which lupus nephropathy (LN) is one and can cause visceral organ failures (Almaani et al., 2017). The EHR data used in the study were from the hospitalization and discharge records of SLE patients from 13 Grade III Level A hospitals in China (Supplementary Table 1). The study was performed according to a protocol approved by the Institutional Committee on Ethics of Biomedicine. There are 14,439 de-identified SLE patients with a total of 57,367 Chinese clinical notes, enrolled from October 28, 2001 to May 31st, 2016. Privacy information, such as patients' name, photo ids, home addresses, and diagnostic dates has been de-identified and anonymized. These SLE patients' averaged diagnosis age is 33.4 years old, including 13,062 females (90.46%). We performed rigorous data quality control by recruiting patients whose EHR recorded the related diagnosis and treatment information corresponding to the time of each admission and discharge. Patients who missed first diagnosis date and only had one visit were excluded from further study. Ultimately, the final data set contains 14,219 patients with 49,752 notes. Among these patients, 14,039 (98.7%) patients have <10 visits.

### Pre-processing to Extract Medical Entities From Unstructured Clinical Notes

**Figure 1:**
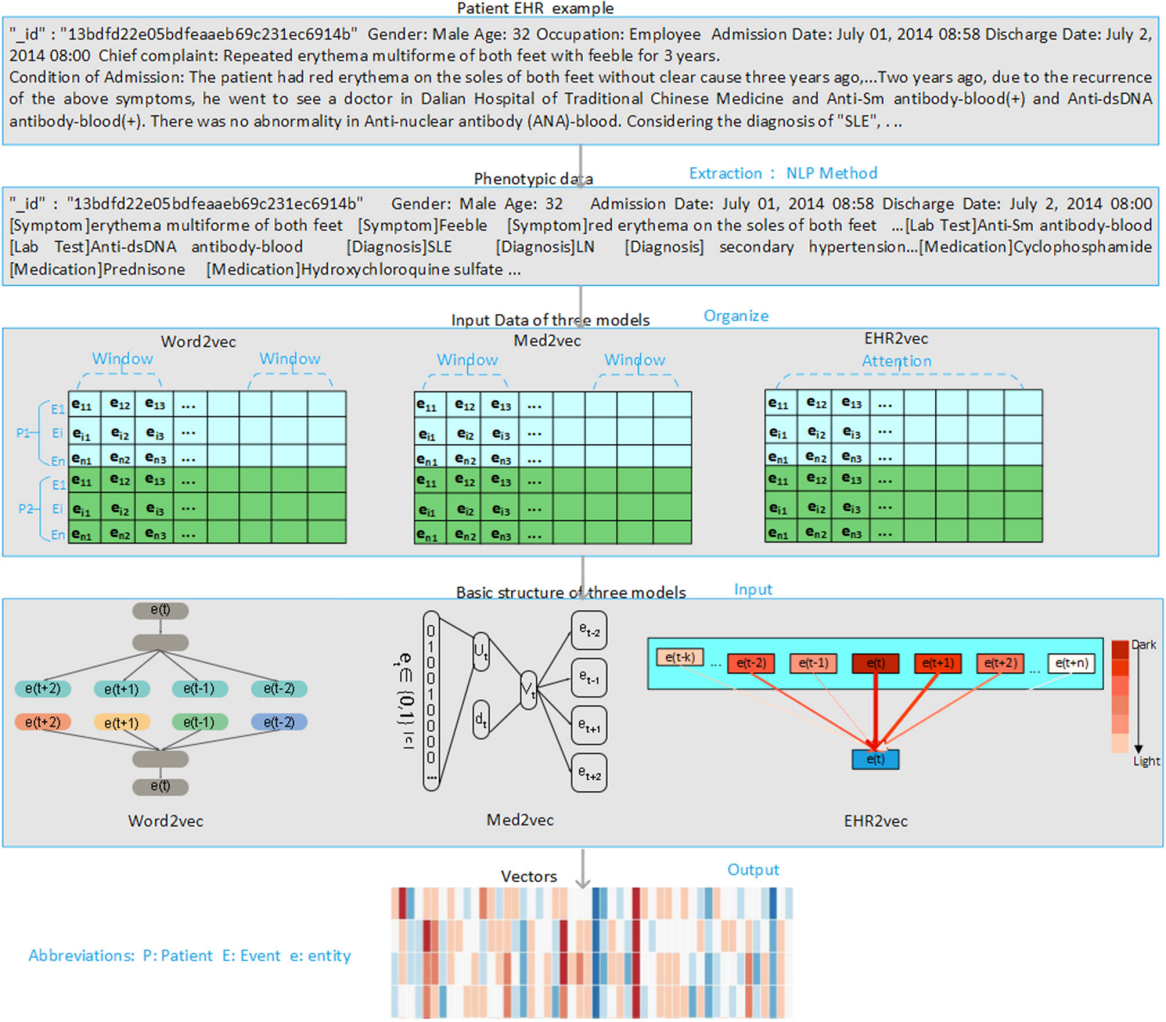
Overview of project design. Our project has four steps. Firstly, we extract medical concepts (i.e., symptoms, lab test, medication, and diagnosis) from free clinical notes of SLE patients' EHR data. Next, we align these medical concepts into structured data format for each note per visit. Then, we sort notes in time series order for each patient. Finally, we comparatively study the performance of three embedding methods: Word2Vec, Med2Vec, and EHR2Vec.

The overview of the EHR2Vec workflow is shown in Figure 1. Medical entities extracted from Chinese clinical notes follow standard pipeline, including word segmentation, part-of-speech tagging, named entity recognition (NER), annotation and normalization using our in-house customized scripts. We collected standardized Chinese medical vocabulary knowledgebase, including thesaurus, grammar, and semantic database, as the prior information for NER task. Word segmentation used a string matching method to identify thesaurus and then used a bi-directional matching method to parse grammar rules. Part-of-speech tagging used a dynamic viterbi algorithm for semantic analysis (Klein and Manning, 2003; Schmid, 2004). Finally, NER task used bi-LSTM algorithm to improve the accuracy and recall continuously. All the medical entities of the patients were tokenized as the input of Word2Vec, Med2Vec, and EHR2Vec for performance comparison.

### EHR2Vec Method for Representations Learning

EHR2Vec has two layers, of which the first is to capture the relations between medical entities within each of the patient's medical event, and the second is to capture the global relations among the different medical visit events. In EHR2Vec, each medical entity within each visiting event undergoes attention computation with the goal of learning the dependencies between medical entity vectors. Assuming each patient *P* had multiple visiting events *E* = {*E*_1_, *E*_2_, …*E*_*n*_}. In a particular visiting event *E*_*i*_, medical entity *j* can be represented as *e*_*ij*_. The medical entities were grouped into four categories for further analysis including symptom, medicine, lab test, and diagnosis. Supplementary Figure 1 illustrated an example of information extraction at different time points and intervals for a patient.

The initialized vector-matrix *W*, is in vector space *R*^h*c^, where *c* is the dimension of each entity vector, *h* is the number of entities in all visits. Here, we used default value *c* = 512, which means each entity maps to 512-dimensional vector space. We first input the patient's initialized vector matrix to the first sublayer (attention mechanism). Equation (1) is the core formula of the attention mechanism that is used, in which Q, K, and V represent the query vector, key vector, and value vector, respectively, and *d*_*k*_ represents the dimension of Q, K, or V. The reason for the division by the square root of *d*_*k*_ is to prevent the product of *QK*^*T*^ from being too large, which may cause the softmax function to enter the saturation region so that the gradient would be too small (Vaswani et al., 2017).

In the model, to extract more features, a multi-head attention structure is adopted, in which a total of eight attention heads are used. Each head can capture different layers of dependency relationships. The eight attention heads are equivalent to eight subtasks, each subtask generating its own attention. The attention calculation of the eight attention heads can be performed through parallel computing to speed up the calculation.

(1) $$\begin{array}{l} \text{Attention}\left(\text{Q},\text{K},\text{V}\right)\;=\text{ softmax}\left(\frac{QK^{T}}{\sqrt{d_{k}}}\right)\text{V} \end{array}$$

### Model Optimization

**Figure 2:**
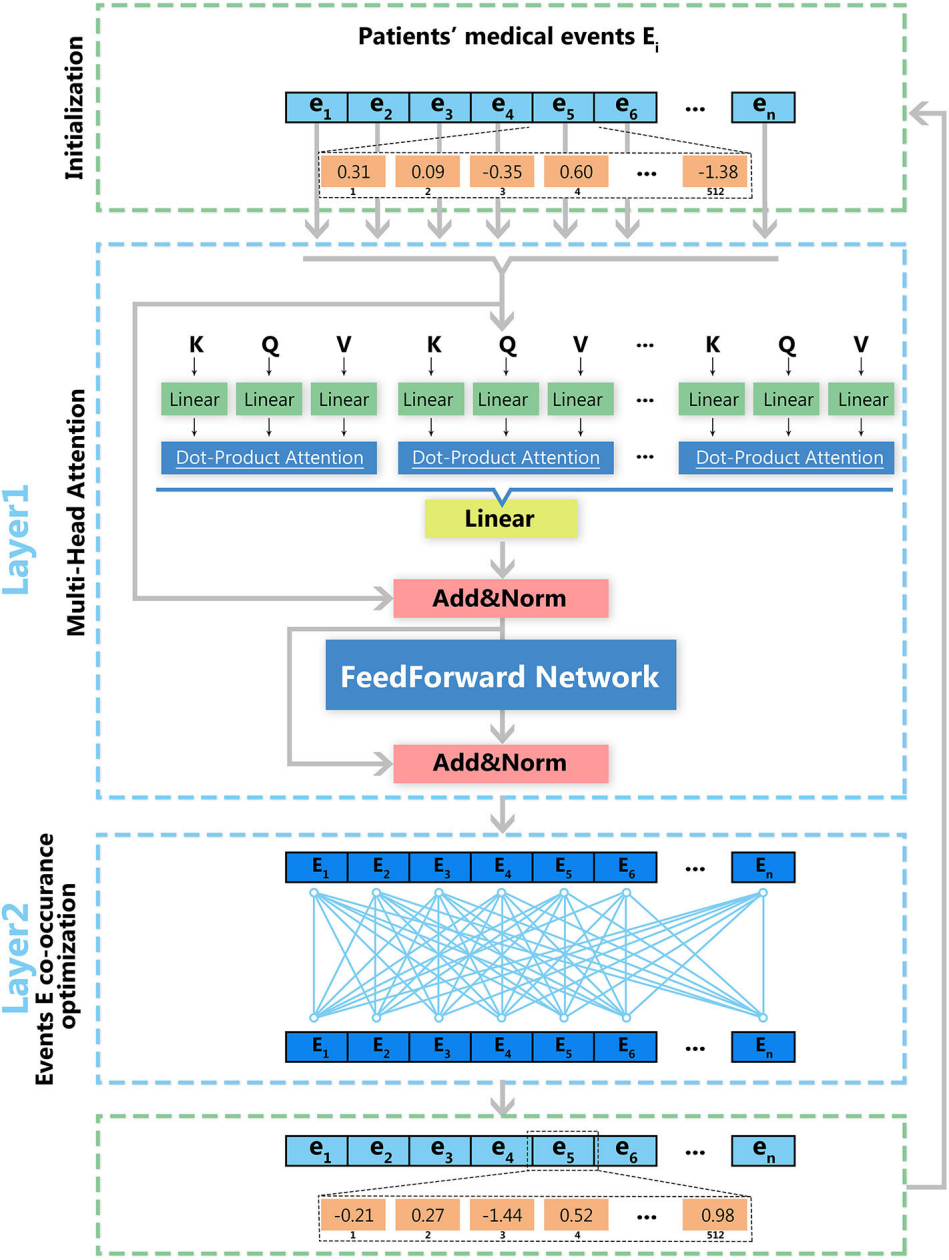
Deep learning architecture of EHR2Vec. EHR2Vec is developed under deep learning framework including two layers of optimizations. The first layer is based on self-attention structure with multi heads to capture the relationship of different medical concepts within each visit event. The second layer is based on co-occurrence of visits to capture the relationships among visits of patients.

As shown in Figure 2, model optimization has two steps. The first step is the optimization for a multi-head attention structure within each visit event. The second step is the optimization of deep feedforward networks for multiple visit events. Vector matrix W is obtained through iterative training, and each row of W represents the vector in the medial entity set. Therefore, we obtain the final medical entity vectors by continuously optimizing the vector-matrix W. The log-likelihood function is used to optimize the obtained medical event vectors as shown in Equation (2), in which *e*_*i*_ and *e*_*j*_ represent medical entities in each medical event and T represents the number of medical events. By maximizing this function's value, we obtain the optimized vector-matrix W.

(2) $$\begin{array}{l} \frac{1}{T}\overset{T}{\underset{t=1}{\sum }}\underset{i:e_{i}\in E_{t}}{\sum }\underset{j:e_{j}\in E_{t},\;j\neq i}{\sum }\log p\left(e_{j}|e_{i}\right)\text{ where},\\ p\left(e_{j}|e_{i}\right)=\frac{\exp\left({W\left[i,:\right]}^{T}W\left[j,:\right]\right)}{\sum_{k=1}^{all}\exp\left({W\left[k,:\right]}^{T}W\left[i,:\right]\right)} \end{array}$$

### Intrusion Analysis for Representations Evaluation

Intrusion analysis is used to test whether the identified representations agree with human judgment. To evaluate the accuracy of EHR2Vec, we performed two types of intrusion analysis (Chang et al., 2009; Murphy et al., 2012; Luo et al., 2015). The first intrusion experiment is to compare with clinical experts' opinions. We calculated the cosine correlation values of a given medical entity's vector against all other medical entities *e*_*ij*_ and then ranked them. We picked the top five medical entities and randomly chosen one medical entity from the last 50% of the ranks, consisting of six medical entities. Clinicians were asked to pick the correct entity set, and the accuracy of the correct choice was calculated using Equation (3), in which $a_{k}^{m}$ represents the *k*th given medical entity in the *m*th model, $i_{k}^{m}$ is the *k*th intrusive medical entity in the *m*th model chosen by the expert, and *S* is the total number of medical entities in the *m*th model.

(3) $$\begin{array}{l} \text{M}P_{k}^{m}=\underset{s}{\sum }1\left(i_{k}^{m}=a_{k}^{m}\right)/S \end{array}$$

The second intrusive experiment is based on the assumption that if a vector accurately captures the relations between the medical entities and patient's medical events, then a certain dimension of the vector will have a certain meaning (Murphy et al., 2012). In order to verify this assumption, we randomly chose several dimensions from the vector result of EHR2Vec, ranked their vector values in descending order and obtained the medical entities corresponding to the first *k* values, as indicated in Equation (4), in which *i* represents the *i*-th dimension, and rank the indices of a vector.

(4) $$\begin{array}{l} \text{argsort}\left(\text{W}\left[:,\text{i}\right]\right)\left[-\text{k}:\right] \end{array}$$

### Implementation and Training Details

EHR2Vec and Med2Vec were implemented and trained using the python TensorFlow 1.8.0 deep learning framework (Abadi et al., 2016). All models were performed on a CentOS server equipped with two 16G NVIDIA TESLA P100 graphics cards. EHR2Vec used the Adadelta optimizer to optimize the target function with a drop rate of 0.1 to achieve model convergence. EHR2Vec used eight attention heads in the self-attention mechanism, and 512 vector dimensions for each entity. To be consistent, the numbers of word vector dimensions of Med2Vec and Word2Vec were also set to 512. The Word2Vec model was implemented by python genism 3.6.0 package, with a window size of 5 and a minimum word frequency of 5. Both EHR2Vec and Med2Vec have trained 20 epochs for the best result.

## Experimental Results and Discussion

### Illustration of Extracted Medical Entities

The statistics of the number of identified NERs can be found in Supplementary Table 2. In details, a total of 10,469 Chinese medical entities, including 1,106 diagnosis entities, 963 medication entities, 8,365 symptom entities, and 35 lab test entities extracted from 49,752 notes, have been translated into English standardized medical vocabularies for results delivery. As the data are shown in Supplementary Table 6, the first column are the de-identified patient IDs, the second column are the de-identified patients' visiting ids, and the rest columns are the example extracted entities.

### Experimental Results Comparing Three Models at a Fixed 512 Vector Dimension

We used LN as the target word in SLE, and calculated its cosine correlation to other medical entity vectors. Cosine distance measures the cosine of the angle between two vectors projected in a high-dimensional space. It is advantageous because even if the two similar medical entities are far apart by the Euclidean disease due to the size of the terms, they may still be oriented closer together. Top 20 medical entities correlated with LN in SLE patients were calculated with medical entities from diagnosis, medication, lab test, and symptom, respectively.

Table 1 showed the comparison of the top 20 medicines associated with LN using EHR2Vec, Word2Vec, and Med2Vec. The top 20 drugs using the EHR2Vec method match the three doctors' medication preference order. For example, the top three drugs, such as hydroxychloroquine sulfate, are the most commonly used hormone prescription for LN treatment. Other drugs, such as Calcium carbonate and vitamin D3 are all auxiliary drugs for the treatment of the related target organ damage and complications, with less relevance. Similarly, Supplementary Tables 3–5 showed the top 20 entities from diagnosis, lab test and symptom. Results from the three methods were also consistent with clinicians' experiences. For example, in the diagnosis results from EHR2Vec, SLE ranked on the top followed by hypertension, lung infection and diabetes, which match with clinician's cognitions. As for the association of pregnancy, one explanation might be that lupus could lead to abnormal birth during pregnancy (Mok et al., 2014).

**Table 1:** Top 20 medication entities with the highest correlation to LN in the vector results obtained using four models.

**Rank**	**Word2Vec**		**Med2Vec**		**EHR2Vec**	
	**Correlation**	**Medication**	**Correlation**	**Medication**	**Correlation**	**Medication**
1	0.68	Albumen	0.31	Tabellae rhei ET natrii bicarbonatis	0.93	Hydroxychloroquine sulfate
2	0.67	Lamivudine	0.31	Ranitidine hydrochloride	0.92	Prednisone acetate
3	0.66	Felodipine	0.27	Iron sucrose	0.90	Methylprednisolone
4	0.66	Cefotaxime	0.25	Terazosin hydrochloride	0.89	Cyclophosphamide
5	0.65	Dexamethasone	0.24	Arotinolol hydrochloride	0.86	Calcium carbonate and vitamin D3
6	0.65	Metoclopramide	0.24	Enalapril maleate	0.82	Omperazole
7	0.65	Dengzhanxixin	0.24	Diammonium glycyrrhizinate	0.79	Calcitriol
8	0.65	Colquhounia root	0.24	Clopidogrel hydrogen sulfate	0.75	Alfacalcidol
9	0.64	Fasudil hydrochloride	0.23	Rabeprazole	0.72	Leflunomide
10	0.63	Salvianolate	0.23	Haloperidol	0.71	Total glucosides of paeony
11	0.63	Thiamazole	0.23	Prednisone	0.70	Aspirin
12	0.62	Cefoperazone Sodium and Tazobactam Sodium	0.23	Levothyroxine sodium	0.65	Prednisolone acetate
13	0.62	Leigongteng	0.23	Lithium carbonate	0.63	Folic acid
14	0.62	Thyroid	0.23	Urokinase	0.62	Levothyroxine sodium
15	0.62	Prednisone	0.22	Penicillins	0.61	Warfarin Sodium
16	0.62	Fluvoxamine maleate	0.22	Carvedilol	0.60	Mycophenolate mofetil
17	0.62	Sodium valproate	0.22	Mecobalamin	0.60	Pantoprazole
18	0.61	Salvianolate	0.21	Furosemide and spironolactone	0.60	Valsartan
19	0.61	Tacrolimus	0.21	Deslanoside	0.58	Spironolactone
20	0.61	Sanqi Panax Notoginseng	0.21	Cefradine	0.57	Low Molecular Weight Heparin Calcium

**Figure 3:**
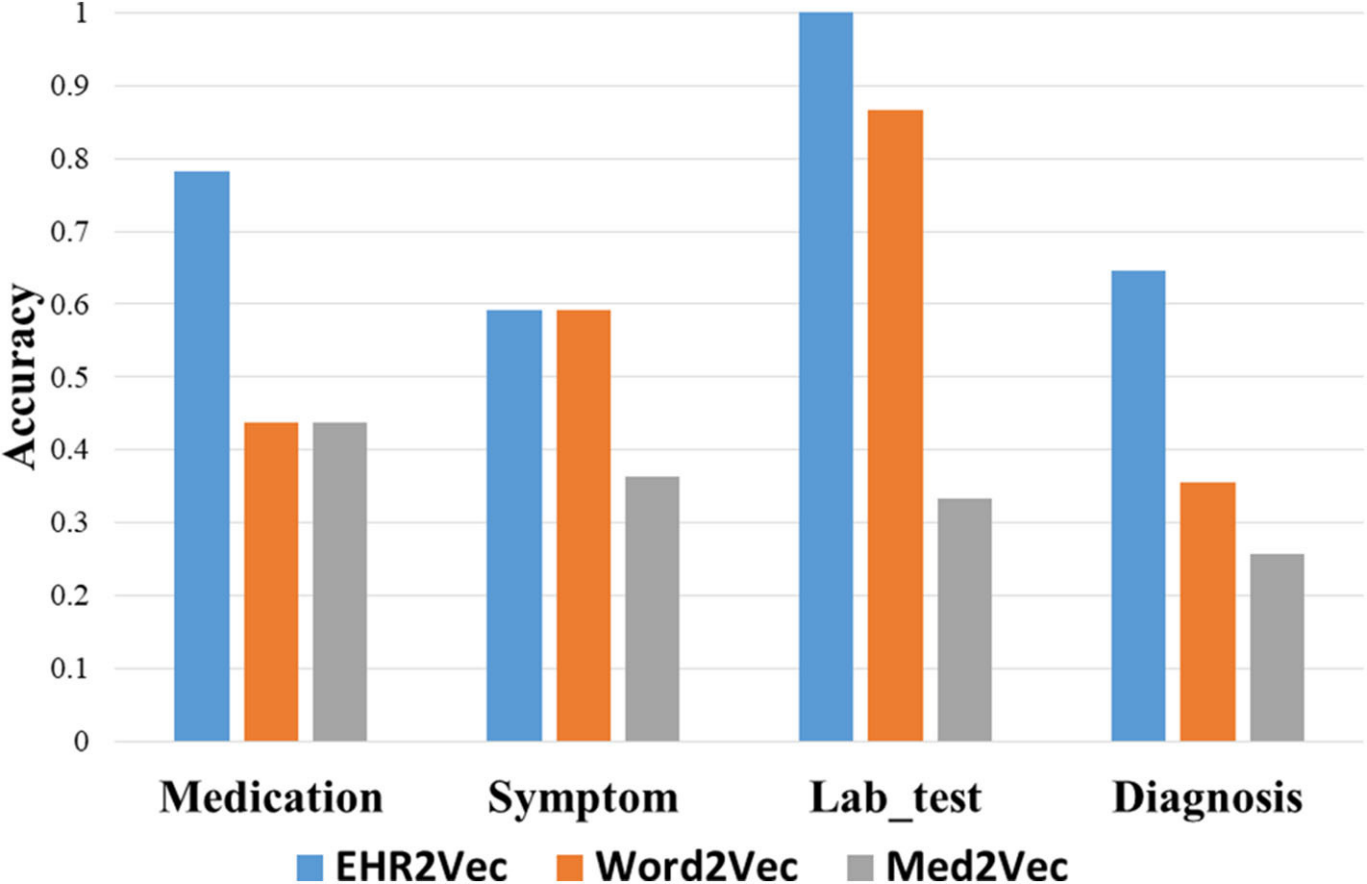
Performance comparison by Intrusion analysis. We perform intrusion analysis to evaluate model performance by comparing clinicians' opinion with our identified medical concepts from four groups. The EHR2Vec shows higher accuracy than the other two models, Word2vec and Med2vec.

However, top rankings from Med2Vec are not consistent with clinical cognitions. For example, the top three ranks in diagnosis from Med2Vec, such as chronic viral hepatitis B, typhoid and fever of unknown origin, were diagnosed poorly related to LN. The top two results of Word2Vec were similar to Med2Vec, but not for the rest. For example, hyperlipemia, hypothyroidism, and fatty liver were considered poorly related to LN. Figure 3 showed a summary comparison of the three models for the four categories. Particularly, EHR2Vec showed over 40% improvement in detecting LN relevant medications in the medicine category.

### Interpretability Evaluation of EHR2Vec Representations at Three Random Dimensions

The results in Table 2 showed the top 10 medical entities at three arbitrarily selected vector dimensions: 180, 274, and 480. Identified entities in the dimension of 180 represented a class of women with a history of pregnancy who suffered from LN, tested positive on the anti-nuclear antibody (ANA)-B and abnormalities on Complement 3-B and Complement 4-B, and used hormone drugs, such as prednisone acetate and methylprednisolone sodium succinate, as well as hydroxychloroquine sulfate. Correlations between these diagnoses, lab tests, and medications are highly consistent with clinical observations. Results from dimension 274 indicated patients who suffered from LN, tested positive on the anti-nuclear antibody (ANA)-B lab test and took commonly used drugs for patients with SLE, such as prednisone acetate and prednisolone sodium succinate while using calcium-supplementary drugs, such as Calcium carbonate and vitamin D3 and gastrointestinal agents, such as omeprazole and aspirin. Dimension 480 represents a number of highly relevant symptoms that often manifest in patients with SLE, including rash, telangiectasia, muscle pain, etc.

**Table 2:** Top 10 medical entities in terms of vector value rank in three different dimensions.

**Dimension 180**	**Dimension 274**	**Dimension 480**
[Lab Test] Complement 3-B	[Medication] Omperazole	[Symptom] Widespread facial red rash
[Diagnosis] Pregnancy	[Lab Test] Urine protein qualitative test-U	[Symptom]Migratory double joint and shoulder pain
[Lab Test] Complement 4-B	[Medication] Calcium carbonate and vitamin D3	[Symptom] Systemic diffusive and red rash
[Drug] Calcium carbonate and vitamin D3	[Medication]Aspirin	[Symptom]Slightly swollen left-hand fingers
[Medication] Methylprednisolone	[Symptom] Cough	[Symptom]Scattered bleeding points on hands
[Medication] Methylprednisolone sodium succinate	[Medication] Methylprednisolone sodium succinate	[Symptom] Facial rash relief
[Lab Test]Anti-nuclear antibody (ANA)-B	[Medication]Prednisone acetate	[Symptom]Capillary and facial capillary expansion
[Medication] Prednisone acetate	[Lab Test]Anti-nuclear antibody (ANA)-B	[Symptom] Muscle and body tenderness
[Diagnosis] LN	[Diagnosis] LN	[Symptom] Scattered red rash
[Medication] Hydroxychloroquine	[Medication] Hydroxychloroquine	[Symptom] Left chest pain

To show the robustness of our interpretable model, we performed another independent experiment by arbitrarily selecting the extra three sets of dimensions: 360, 440, 457. We can see the results in Supplementary Table 7, dimension 360 is more related to manifestations of the mucous membrane of the skin, such as subcutaneous bleeding, facial rash, palm erythema, and oral ulcer; dimension 457 is more related to appearance symptoms, such as phenotypes in face, skin, ulcers, etc.; dimension 440 is more associated with vasculitis, such as hypertension, edema, rash, and erythema. All the above evidence indicated the interpretability of our model by showing that the top medical entities in each dimension are highly relevant.

### SLE Disease Comorbidity Prevalence and Progression Over Time

**Figure 4:**
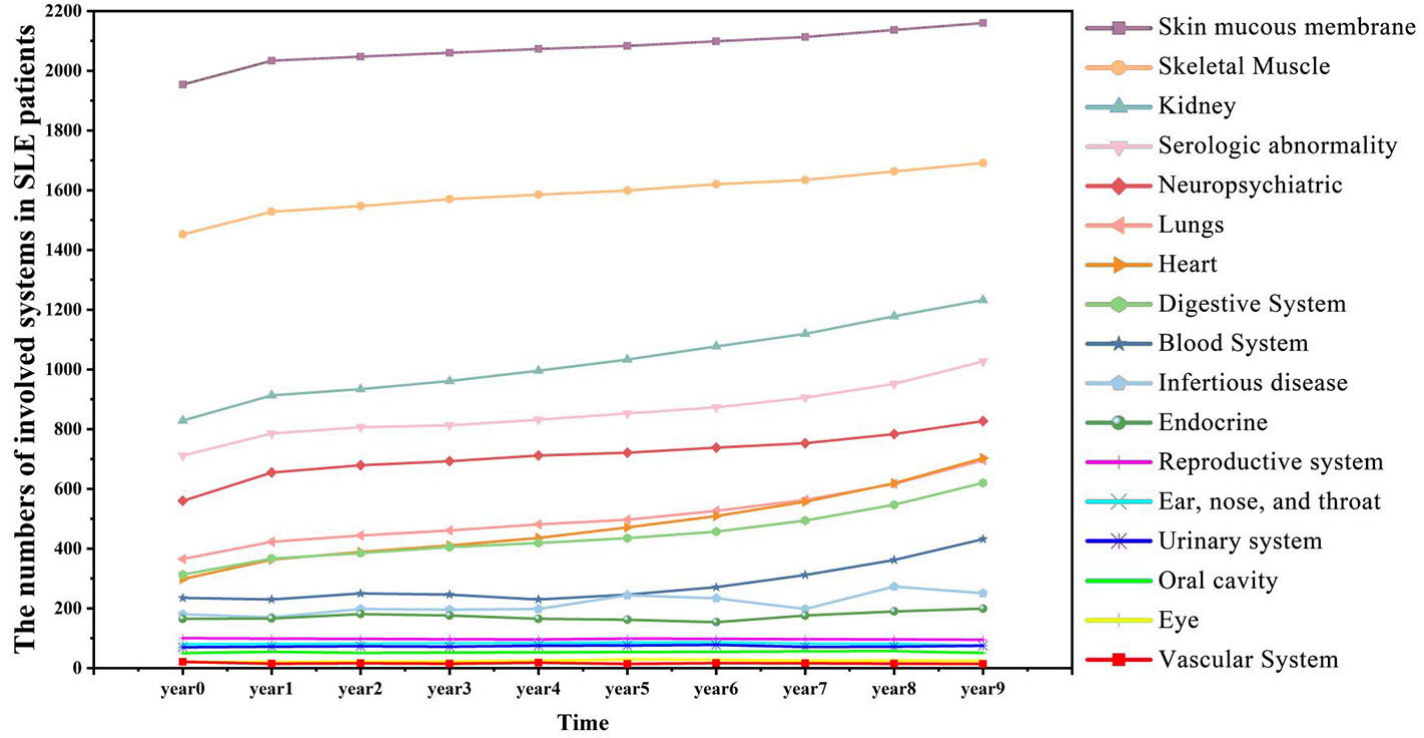
SLE affects more patients' body organs and systems over time. SLE is a chronic disease with many comorbid conditions. This figure shows that more SLE patients are affected by comorbidities as the time accumulated. For example, in the initial year (Year 0), 829 patients manifested kidney diseases, and in Year 9, the number increased to 1,232.

Figure 4 showed a summary of the prevalence of SLE comorbidity changes over time diagnosed by clinicians. The comorbidities of SLE diagnosed by clinicians are correlated with medical entities, such as symptoms and diagnosis ranked by EHR2Vec. For example, skin mucous membrane lesions (SMML) is the most prevalent comorbidity than any others. The number of this group of patients is gradually progression year by year. Some skin related symptoms, such as rash are also ranked by top candidate entities related to LN from EHR2Vec analysis, which has a high agreement with clinician's diagnosis. While some of the comorbidities, such as blood system, are more complex in disease progression. For example, no or little progression in the first 5 years, but dramatically increased after 5 years. These types of comorbidities' symptoms may have challenges to be detected by EHR2Vec, which requires further validation by clinicians. Nevertheless, existing evidence showed that EHR2Vec is able to rank the most relevant disease-related phenotypic information from raw EHR data automatically through key word query.

## Discussion

EHR data with sequential visiting records provides opportunities for disease early detection. Automatically extracting medical concepts from EHR data and converting them into embedding vectors can contribute significantly to monitor the conditions change for chronic disease. In this project, we developed a new embedding method, EHR2Vec, to learn representative medical concepts from EHR data. EHR2Vec incorporated self-attention algorithm with multi-layer deep learning optimizations at both medical codes and visits level. EHR2Vec overcomes existing embedding methods that ignored contextual information at visit level or missed multi-visit information to capture temporal patterns from clinical notes. In the experiments of SLE data, one of a chronic disease, EHR2Vec has displayed its significant improvement in key medical applications, while providing clinically meaningful interpretations. Using EHR2Vec to learn representations will improve the accuracy for detecting disease prevalence and progression in precision medicine.

Traditional NLP word embedding approaches, such as Word2Vec published in 2013, are unable to capture long-distant dependency relations for words in a sentence, here medical entities in a visiting event. Later, improved architectures RNN-based Bi-LSTM method, such as ElMo (Peters et al., 2018), were developed to capture contextualized information in time order, however its computational efficiency is less optimal in identifying long-range dependency since it has to remember all the state of words sequence by sequence and disallowance of the parallel computing in a long sequence. Recently, self-attention based algorithms overcome those limitations and has become the leading architecture in NLP tasks since 2018. It is the key component utilized in many state-of-art transformer learning methods, such as BERT (Devlin et al., 2018), GTP-2 (Radford et al., 2019), and XLnet (Yang et al., 2019), allowing to identify long-range relationships that are far away through parallel computation. By applying self-attention structure in real-world EHR data, our EHR2Vec optimized both sequential information from different visits and different types of medical entity relations within each visit, which greatly improves traditional methods in representation learning for relevant comorbidity detection in chronic disease.

EHR2Vec showed great performance in comparison with the other two most popular representation learning methods in EHR data. We queried a medical term, LN, which is a common comorbidity in SLE patients. The experimental results of EHR2Vec from medication category indicated that the medication preference has the highest correlation with LN. While the logical sequence was chaotic and the frequencies of the features were small with no representativeness in Word2Vec or Med2Vec method. In the Word2Vec method, the vector of the center word is obtained by simply summing the vector values of the context words, so the generated vector has an indistinctive boundary, causing heterogeneous medical concept ambiguities. The Med2Vec model also has a fixed window size, leading to poor word vector discriminations. EHR2Vec solves the problem by self-attention structure to capture global information, thus the concept representation vector calculated is more accurate while allowing further optimization on the medical event sequence of each patient, thereby leading to higher discrimination of the final medical entity vector.

We also recognize that there are some areas that can be further improved in EHR2Vec in the future. First, although we optimized the visiting events at the time orders for each patient's EHR data, some of the time sequence information within each visit might be poorly extracted and captured. Second, a more comprehensive propagation networking algorithm could be combined with self-attention structure to quickly target information of importance for each event. Nevertheless, these tasks are the most challenging part for the clinical NLP in every EHR data and more accurate models can be developed according to specific scenarios in the real-world situation. Finally, we only evaluated a large-scale SLE dataset in the current study, but we plan to expand to other disease areas where clinical symptoms and comorbidity change over time, such as autism spectrum disorders, to assess the generalizability of the proposed approach in the future.

## Conclusion

In this study, we proposed the EHR2Vec model, a new deep learning model that generates medical entity vectors based on the attention mechanism. Compared with other widely used word vector generation models (e.g., Word2Vec and Med2Vec), EHR2Vec can correct target medical entity more accurately using self-attention structure to capture relations from surrounding medical entities. We compared and tested the performance of EHR2Vec through clinical expert assessments and an intrusion experiment using the SLE dataset, an actual clinical disease dataset, and found that EHR2Vec could generate more accurate vectors. In the future, we will integrate more medical knowledge (e.g., doctors' prior knowledge and patient image data) into the EHR2Vec model and apply the resulting vector to more scenarios, e.g., SLE complication and hospitalization length predictions.

## Data Availability Statement

EHR2Vec code can be found: https://github.com/jingsongs/EHR2Vec.

## Ethics Statement

The studies involving human participants were reviewed and approved by Committee on Ethics of Biomedicine, Second Military Medical University. The Ethics Committee waived the requirement of written informed consent for participation.

## Author Contributions

LW and QW contributed on the project design and code implementation. HB, WL, and YZha contributed on the EHR database management and data curation. LJ and HX performed the statistic analysis. YZho and KW contributed on the project design and supervised the project. All authors contributed to the article and approved the submitted version.

## Conflict of Interest

The authors declare that the research was conducted in the absence of any commercial or financial relationships that could be construed as a potential conflict of interest.
