# Supplementary figures and images for: EHR2Vec: Representation Learning of Medical Concepts From Temporal Patterns of Clinical Notes Based on Self-Attention Mechanism
Source: Front Genet. 2020 Jun 29;11:630. doi: 10.3389/fgene.2020.00630 (PMC7344186; doi:10.3389/fgene.2020.00630)

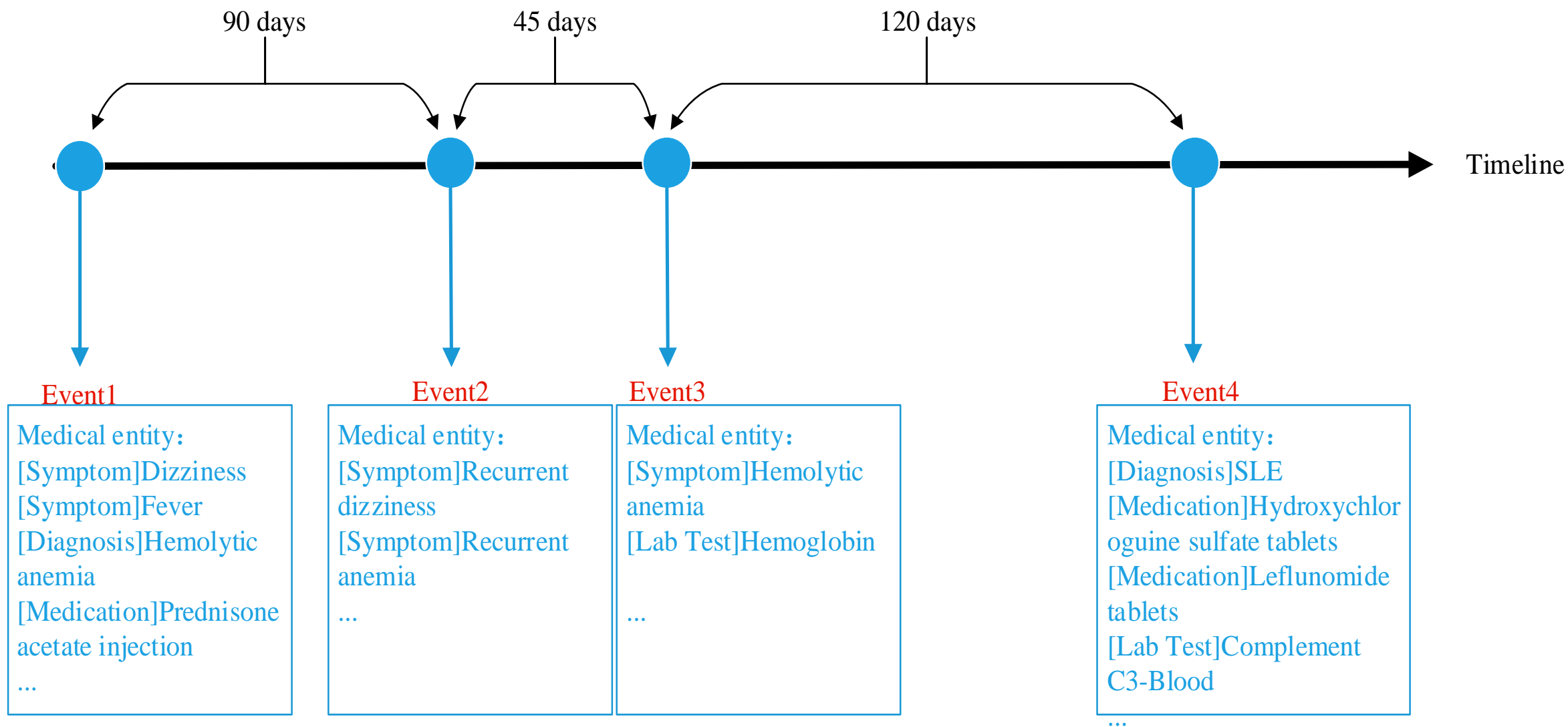

Supplement: Supplementary file 2 [file Image_1.pdf]
